# Changes in tumor necrosis factor-a (TNF-a), interleukin-6 (IL-6), matrix metallopeptidase-2/9 (MMP-2/9), and T-lymphocyte subsets in patients undergoing thoracoscopic surgery under refined management for non-small-cell lung cancer

**DOI:** 10.5937/jomb0-51316

**Published:** 2025-03-21

**Authors:** Shuxin Cai, Yannan Zhang

**Affiliations:** 1 Affiliated Hospital of Nantong University, Department of Thoracic Surgery, Nantong, China

**Keywords:** non-small-cell lung cancer, thoracoscopic surgery, refined nursing, inflammatory response, t lymphocyte subsets, rak pluća ne-malih ćelija, torakoskopska hirurgija, poboljšana nega, inflamatorni odgovor, T-limfocitne subpopulacije

## Abstract

**Background:**

Non-small-cell lung cancer (NSCLC) remains one of the most common malignancies worldwide, posing a great potential threat to the health and life safety of patients.

**Methods:**

One hundred and fifty NSCLC patients admitted to our hospital from September 2022 to September 2023 were selected whose inflammatory response and changes in T-lymphocyte subpopulations before and after surgery were detected, and patients' psychological status was investigated. Finally, patients were followed up for 6 months to record the prognostic survival rate.

**Results:**

Postoperative inflammatory factors and CD8 + were lower in the research group than in the control group, while CD3 + , CD4 + and CD4 + /CD8 + were higher than in the control group (P<0.05). Meanwhile, the psychological status of the research group was better. However, the two groups did not differ in prognostic survival (P>0.05).

**Conclusions:**

Perioperative refinement of care can optimize the surgical process in patients with NSCLC, and the cellular immune function was enhanced.

## Abstract (sr)

**Uvod:**

Rak pluća ne-malih ćelija (NSCLC) je i dalje jedna od najčešćih malignih bolesti u svetu i predstavlja veliku potencijalnu pretnju po zdravlje i 'životnu bezbednost pacijenata.

**Metode:**

Izabrano je sto pedeset pacijenata sa NSCLC, primljenih u našu bolnicu od septembra 2022. do septembra 2023. godine, kod kojih su detektovani inflamatorni odgovor i promene u subpopulacijama T-limfocita pre i nakon operacije, a istraživano je i psihološko stanje pacijenata. Na kraju su pacijenti praćeni tokom 6 meseci kako bi se zabeležila prognozirana stopa preživljavanja.

**Rezultati:**

Postoperativni inflamatorni faktori i CD8+ su bili niži u grupi koja je ispitivana nego u kontrolnoj grupi, dok su CD3+, CD4+ i CD4+/CD8+ bili viši nego u kontrolnoj grupi (P0.05).

**Zaključak:**

Perioperativno poboljšanje nege može optimizovati hirurški proces kod pacijenata sa NSCLC, a imunska funkcija ćelija je poboljšana.

## Introduction

Lung cancer (LC), one of the most common malignancies globally, occurs in approximately 51 per 100,000 people, with more than 700,000 new cases worldwide each year [1]. Meanwhile, LC is the leading cause of cancer-related deaths, with approximately 1.4 million patients dying from LC each year and a 5-year survival rate of less than 16% [2]. Non-smallcell lung cancer (NSCLC) is the most common pathological type of LC, accounting for more than 85% of all LC cases [3]. Compared with small-cell LC, NSCLC is associated with a higher probability of cure because it has slower growth and division of cancer cells and a lower risk of diffusion and metastasis [4]. Thoracoscopic radical surgery is the most direct treatment for NSCLC. However, mechanical invasive procedures and partial lung tissue resection cause significant stress trauma to patients, increasing the risk of postoperative complications and infections, the key factors affecting patient recovery [5]. Presently, it is widely recognized in clinical practice that the exacerbation of inflammation and immune dysfunction following NSCLC surgery are unavoidable adverse events [6], and how to control these adverse conditions has become a hotspot in modern clinical research.

A high-quality surgery often requires a comprehensive, meticulous, and high-quality perioperative nursing model, and vice versa; high-quality perioperative nursing is a powerful guarantee for normal perioperative operation and smooth surgery [7]. The research shows that in laryngeal cancer, knee replacement, and other surgeries, the new care model of perioperative refined nursing can improve the quality of care and surgical effect while providing more reliable protection for patients with postoperative stress injuries by making the nursing links in the perioperative meticulous and high-quality [8] [9]. However, for NSCLC, there is still a lack of reliable references for perioperative individualized care.

Based on this, this study explores the influence of perioperative refined nursing care on T lymphocyte subsets and inflammatory responses in patients undergoing thoracoscopic surgery for NSCLC to provide new reference and guidance for future surgical care of NSCLC patients and ensure their prognoses.

## Materials and methods

### Study subjects

One hundred and fifty NSCLC patients admitted to our hospital from September 2022 to September 2023 were selected. All patients underwent thoracoscopic surgery in our hospital and were randomized to a control group (n=75) receiving perioperative routine care and a research group (n=75) receiving perioperative refined care using a random number table. This study has been approved by the Ethics Committee of our hospital (Approval No: 2022-L144), and all subjects signed an informed consent form. In addition, we will strictly follow the Declaration of Helsinki when conducting the study.

### Criteria for patient enrollment and exclusion

Inclusion criteria: (1) Meeting the LC diagnostic criteria [10], with NSCLC confirmed by pathological examination; (2) TNM stage [11]: I-III; (3) Preoperative examination showed no metastasis; (4) In line with the indications for thoracoscopic radical surgery for LC [12]. Exclusion criteria: (1) Inability to resect the tumour at one time or presence of distant metastasis; (2) Major diseases such as circulation, blood, and psychiatric diseases; (3) Respiratory failure and pulmonary infection; (4) Conversion from thoracoscopic LC radical surgery to thoracotomy; (5) Estimated survival < 6 months.

### Surgical methods

All patients underwent surgery performed by the same surgical team at our hospital. The procedure involved positioning the patient in the lateral decubitus position and administering general anaesthesia to insert a double-lumen endotracheal tube. Subsequently, a 3.5–5.0 cm incision was made in the 4th or 5th intercostal space along the anterior axillary line. An elastic rubber protective sleeve, thoracoscope, and operating instruments were introduced through this incision. Guided by the thoracoscope monitor, the lung lobes were excised, and the mediastinal and hilar lymph nodes were meticulously cleaned. After inserting a drainage tube (which remained in place for 24–48 hours), the chest cavity was closed, and the incision was sutured [13].

### Nursing methods

### Control group:

The surgical ward nurse thoroughly reviewed the patient’s surgical information, assisted the anesthesiologist with tracheal intubation, anaesthesia induction, and other procedures, ensured that the deep venous access remained unobstructed, and helped the patient maintain the surgical position. Additionally, the nurse prepared surgical instruments and devices, collaborating with the surgeon during incision, puncture, flushing, and other surgical operations. The patient’s vital signs were closely monitored throughout the procedure.

### Research group:

Preoperative care: Instruct the patient to cease smoking at least two weeks before surgery. Prevent and control infections, maintain oral hygiene, and follow the doctor’s instructions regarding antibiotics and nebulized drugs. Strengthen nutritional support to improve the patient’s nutritional status and enhance resistance. Teach the patient abdominal breathing exercises, which can be practised in lying, sitting, and standing positions.

*Intraoperative care: *Prohibit unnecessary conversation during surgery and minimize noise interference. Avoid pushing or pulling the patient when moving them; assist the anesthesiologist in adjusting the patient’s position after general anaesthesia to maintain the correct surgical posture. Select a new type of chest cushion with moderate height, and secure the patient’s knee joint and hip with a soft strap to prevent excessive bending. Place a headband around the patient’s head to avoid head suspension. Apply lidocaine cream to the urinary catheter to minimize postoperative discomfort during urination. Position the thoracoscopic display at the head end, set up the surgical parameters, and perform image acquisition and saving as required during the operation. Monitor the patient’s vital signs throughout the surgery, ensuring that no instruments press against the patient and that thin therapeutic towels are placed around pressure points on the skin. Keep peripheral veins open and ready for resuscitation. Instrument nurses will assist the surgeon by connecting the fixed wires and soaking the lens in warm water in advance to avoid fogging, ensuring a clear surgical field. Strictly adhere to aseptic techniques and pass instruments according to the surgeon’s preferences. To prevent blood contamination of the incision, place a surgical towel near the incision in advance and carefully check all instruments before concluding the operation.

*Postoperative care:* Closely monitor and record changes in vital signs. Choose the appropriate bedside position based on the patient’s surgical situation and physical condition (e.g., flat position when waking from anaesthesia, with the head tilted to one side, or a low semi-recumbent position once awake after surgery). Maintain airway patency, provide oxygen therapy, assist the patient in turning every 1–2 hours, and perform back-patting to promote sputum expulsion. Ensure smooth chest drainage; after total pneumonectomy, the drainage tube is generally clamped in a closed state, with q2h open drainage for 10–15 minutes, ensuring the liquid discharge rate does not exceed 100 mL each time. Six hours after surgery, allow the patient to drink based on intestinal peristalsis. The diet should begin with fluid or semi-fluid options, emphasizing highprotein, high-calorie, vitamin-rich, and easy-to-digest foods. Evaluate the patient’s psychological state and, if necessary, administer sedatives or sleep aids as prescribed for those experiencing excessive anxiety or insomnia. Encourage patients to express their feelings and establish a positive nurse-patient relationship to foster a constructive mindset.

*Complications treatment:* Implement targeted pre ventive measures for common complications following LC surgery. Regularly check the wound dressing and the area around the drainage tube for blood seepage, and monitor the colour and character of pleural fluid to prevent intrathoracic haemorrhage. Watch for signs of tachycardia, elevated temperature, croup, cyanosis, and respiratory distress to prevent lung infection or pulmonary atelectasis. Immediately address any adverse symptoms, providing feedback to the attending physician and assisting with targeted treatment.

*Postoperative health guidance:* Remind patients and their families to focus on smoking cessation,avoid passive smoking, attend regular checkups, and strengthen nutritional support. Provide guidance on postoperative rehabilitation training, such as elevating the head of the patient’s bed by 30–45° and having family members stabilize the patient’s chest with both hands to reduce pain. Instruct the patient to take 5–6 deep breaths, hold their breath after deep inhalation with tightly closed vocal folds, and then cough several times to bring sputum near the pharynx. Finally, encourage a forceful cough to expel the sputum.

### Prognostic follow-up

A 6-month prognosis follow-up was performed on all patients through regular follow-ups. The prognostic survival of patients was recorded, and the survival curves were drawn. At the final follow-up, patients’ quality of life was assessed using the MOS item short-form health survey (SF-36) [14]. The SF-36 consists of eight dimensions: Physical Functioning, Role-Physical, Bodily Pain, General Health, Vitality, Social Functioning, Role-Emotional, and Mental Health, with higher scores indicating a better quality of life for patients.

### Outcome measures

(1) Baseline data such as patient age, sex, operation time, intraoperative blood loss, drainage tube indwelling time, and postoperative complications were analyzed. (2) Fasting venous blood was collected before and 3 days after surgery and divided into two parts: one was detected by enzyme-linked immunosorbent assay (ELISA) for tumour necrosis factor-α (TNF-α), interleukin-6 (IL-6), and matrix metallopeptidase-2/9 (MMP-2/9), and the other was measured by a flow cytometer for CD^3+^, CD^4+^, and CD^8+^ of T lymphocyte subsets, with the CD^4+^/CD^8+^ ratio calculated. (3) The Hamilton Anxiety (HAMA) and Depression Scale (HAMD) [15] were used to evaluate patients’ psychological status before and 7 days after surgery; higher scores suggest more serious negative emotions. (4) The prognostic survival of the two groups was analyzed.

### Statistical analysis

This study used SPSS26.0 for statistical analysis. Count data were recorded as [n(%)] and analyzed with chi-square tests. Measurement data were recorded as (x̄±s), and t-tests were used for comparison. Patient survival was calculated and compared using the Kaplan-Meier and Log-rank tests, respectively. We considered P<0.05 statistically significant.

## Results

### Comparison of clinical data

Table 1 shows that the two groups were similar in age, sex, course of disease, histopathological type, etc. (P>0.05).

**Table 1:** There was no difference in clinical data between the two groups.

Data	Control group<br>(n=75)	Research group<br>(n=75)	χ^2^ (or t)	P
Male	46 (61.33)	50 (66.67)	0.463	0.496
Female	29 (38.67)	25 (33.33)		
Squamous carcinoma	26 (34.67)	22 (29.33)	0.758	0.685
Adenocarcinoma	48 (64.00)	51 (68.00)		
Large cell carcinoma	1 (1.33)	2 (2.67)		
TNM Stage I	34 (45.33)	27 (36.00)	1.378	0.502
TNM Stage II	33 (44.00)	38 (50.67)		
TNM Stage III	8 (10.67)	10 (13.33)		
Age	65.12±5.62	66.63±5.33	1.684	0.094

### Comparison of surgical conditions

**Figure 1:**
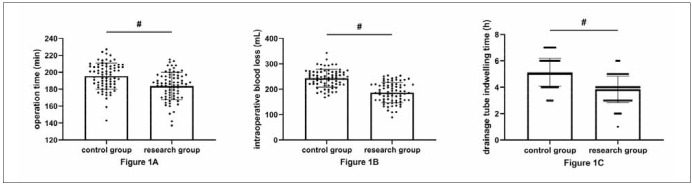
The research group had a superior surgical profile.<br>A: Comparison of surgical time. B: Comparison of intraoperative bleeding. C: Comparison of drainage tube indwelling time.

As shown in Figure 1, the operation time, intraoperative blood loss, and drainage tube indwelling time were all statistically shorter in the research group compared with the control group (P<0.05).

### Comparison of safety

As shown in Table 2, the incidence rates of postoperative complications in the research and control groups were 20% and 34%, respectively, with a markedly lower incidence in the research group (P<0.05).

**Table 2:** Lower complication rates in the research group.

Groups	n	Pulmonary<br>atelectasis	Pulmonary<br>edema	Infection	Hypoxemia	Severe<br>pain	Overall<br>incidence
control	75	3 (4.00)	4 (5.33)	3 (4.00)	5 (6.67)	4 (5.33)	19 (25.33)
research	75	2 (2.67)	2 (2.67)	1 (1.33)	1 (1.33)	3 (4.00)	9 (12.00)
χ^2^							4.391
P							0.036

### Comparison of inflammatory responses

**Figure 2:**
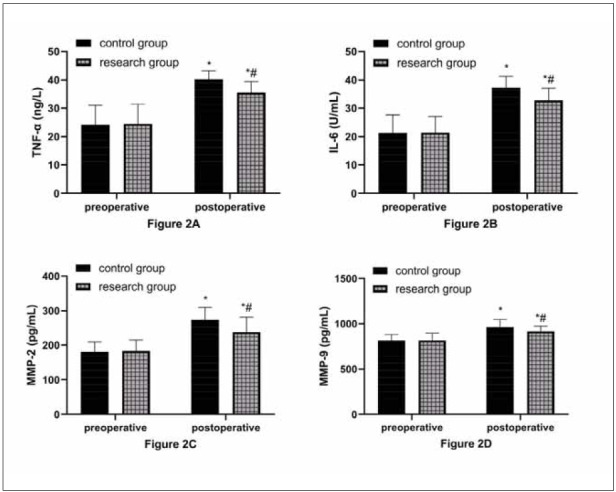
Postoperative inflammatory response was lower in the research group.<br>A: Comparison of TNF-α. B: Comparison of IL-6. C: Comparison of MMP-2. D: Comparison of MMP-9. ^*^ and ^#^ indicate statistically significant differences from the preoperative and control group, respectively (P<0.05).

As shown in Figure 2, the two groups did not differ in preoperative inflammatory factor levels (P>0.05). After surgery, TNF-α, IL-6, MMP-2, and MMP-9 in both groups increased, but with lower levels in the research group compared with the control group (P<0.05).

### Comparison of immune function

**Figure 3:**
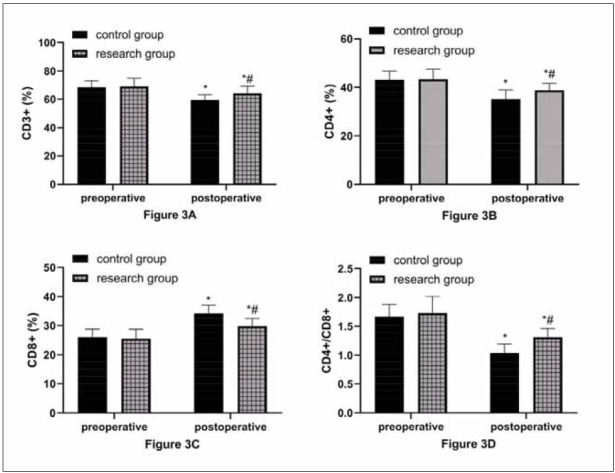
Better postoperative immune function in the research group.<br>A: Comparison of CD^3+^. B: Comparison of CD^4+^. C: Comparison of CD^8+^. D: Comparison of CD^4+^/CD^8+^. ^*^ and ^#^ indicate statistically significant differences between the preoperative and control groups (P<0.05).

As shown in Figure 3, the two groups did not differ statistically in preoperative T lymphocyte subsets (P>0.05). Postoperatively, CD^3+^, CD^4+^, and CD^4+^/CD^8+^ in both groups decreased, but their levels were still higher in the research group versus the control group; CD^8+^ increased in both groups after surgery and was even higher in the research group, which was lower in the research group than in the control group (P<0.05).

### Comparison of psychological status

**Figure 4:**
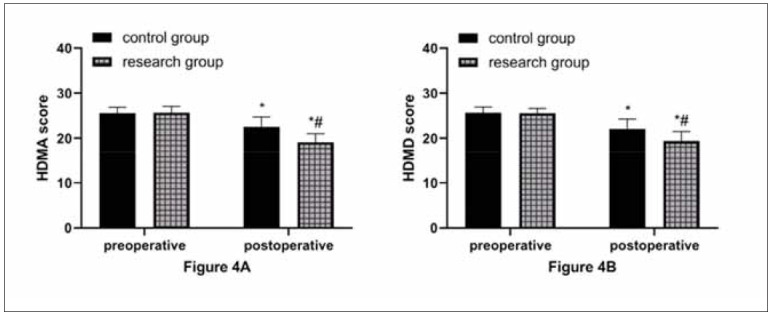
Better postoperative psychological status in the research group.<br>A: Comparison of HDMA score. B: Comparison of HAMD score. ^*^ and ^#^ indicate statistically significant differences between the preoperative and control groups (P<0.05).

As shown in Figure 4, HDMA and HAMD scores were not statistically different between groups before surgery (P>0.05). An obvious reduction in HDMA and HAMD scores was observed in both groups after surgery, with more significant decreases in the research group (P<0.05).

### Comparison of prognosis

**Figure 5:**
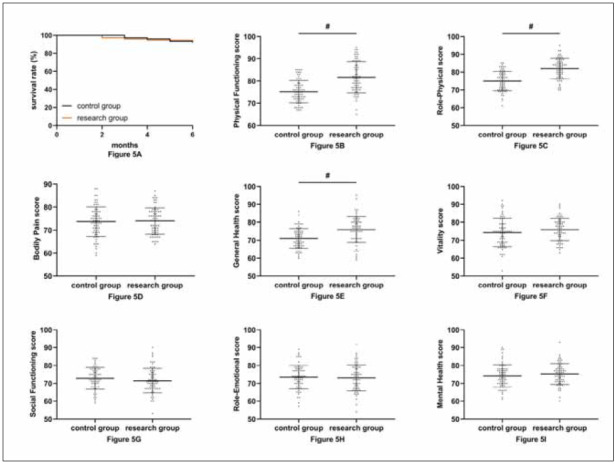
Comparison of prognosis.<br>A: No difference in prognostic 6-month survival between the two groups. B: Comparison of Physical Functioning score. C: Comparison of Role-Physical score. D: Comparison of Bodily Pain score. E: Comparison of General Health score. F: Comparison of Vitality score. G: Comparison of Social Functioning score. H: Comparison of Role-Emotional score. I: Comparison of Mental Health score. ^#^ indicate statistically significant differences from the control group (P<0.05).

As shown in Figure 5, the two groups did not differ significantly in prognostic survival (P>0.05). The results of SF-36 scores showed that there was no difference in the comparison of Bodily Pain, Vitality, Social Functioning, Role-Emotional, and Mental Health scores between the two groups (P>0.05). However, Physical Functioning, Role-Physical, and General Health scores were higher in the research group than in the control group (P<0.05).

## Discussion

Surgery remains the primary treatment for all malignancies, including NSCLC. Managing postoperative stress damage and mitigating inflammation is crucial in ensuring favourable patient outcomes [16] [17]. In this study, we explore the application value of refined care in thoracoscopic surgery for NSCLC, which can provide a reliable reference for the future treatment of NSCLC.

First of all, we compared patients’ surgical outcomes. We found significantly shortened operation time, intraoperative blood loss, and drainage tube indwelling time in the research group using perioperative refined care compared with the control group using perioperative routine care, suggesting that refined nursing helps optimize thoracoscopic surgery and reduce intraoperative injuries in patients. According to previous research reports, refined nursing for lung volume reduction surgery and breast cancer surgery can significantly accelerate the postoperative rehabilitation cycle of patients [18] [19]. The reason may be that perioperative refined nursing is more meticulous based on routine nursing, paying attention to nursing details, improving the overall quality of nursing, and preventing adverse events in advance from patients’ psychological and physiological aspects, thus improving surgical safety and quality [20].

In the subsequent detection of inflammatory responses and T lymphocyte subsets, it was found that TNF-α, IL-6, MMP-2, MMP-9, and CD8+ in the two groups increased after surgery, while CD3+, CD4+, and CD4+/CD8+ decreased, confirming intensified inflammatory reactions in both groups after the operation and the seriously damaged immune function. This is mainly due to the pressure and friction of surgical instruments on the intercostal nerves and the stress response after organ and tissue resection [21]. In the inter-group comparison, the inflammatory factors were lower in the research group versus the control group. At the same time, the stability of T lymphocyte subsets was higher, indicating that refined care is also conducive to relieving the stress injury of patients after surgery, consistent with the research results of Su H et al. [22]. This is because the catabolism of NSCLC patients is greater than the anabolism. Acute proteins are consumed in large quantities, making it challenging to meet the supply capacity required for T lymphocyte proliferation [23]. In addition, surgery-induced stress injury will aggravate this malignant state, which will lead to the continuous decline of patients’ immunity and the accelerated release of inflammatory mediators, eventually leading to various complications and infections [24]. Under refined nursing, the patients’ perioperative comfort has been effectively improved, and the risk of complications has been avoided by paying attention to their adverse state during the perioperative period and giving effective counselling. Besides, reasonable healthy diet guidance can also greatly ensure patients’ basic health, laying the foundation for their postoperative recovery.

Furthermore, postoperative rehabilitation training and other guidance can speed up the transfer of secretions from small airways to large airways, promote the discharge of secretions from the body, avoid airway obstruction, reduce the damage to patients’ lung function, and improve the contractility of respiratory muscles [25]. This is also conducive to inhibiting the release of inflammatory factors and improving patients’ immune function recovery. Among them, MMP-2 and MMP-9 belong to the MMP family, which has been regarded as an excellent inflammatory factor in recent years and has been involved in developing many diseases [26] [27]. We chose them as inflammatory response indicators, which is not only novel, but their close relationship with NSCLC can help us more accurately determine patients’ pathological changes after surgery [28] [29].

Moreover, in comparing psychological status, the lower HDMA and HAMD scores in the research group versus the control group suggest that refined nursing has a better positive effect on patients’ psychological status. We believe that refined nursing can achieve close connections of various nursing links and make the operation more controllable, accurate, and reasonable, with its core concept lying in precision, fineness, meticulousness, and strictness. This kind of nursing appropriately adjusts the psychological and physical status of LC patients undergoing thoracoscopic radical surgery, strengthens the calming of patients’ treatment emotions, and reduces the pressure of rehabilitation based on basic nursing work to avoid adverse psychology’s impact on disease treatment. There is undoubtedly potential for optimization and enhancement in patient care. Specifically, there is a need to bolster health education regarding disease awareness and to enhance patients’ comprehension of NSCLC.

Additionally, improving the nursing capabilities of nurses is imperative to guarantee the delivery of the utmost professional and precise nursing care to each patient. However, the two groups had no significant difference in the prognostic survival rate. It is speculated that this is due to the short follow-up time and the fact that the subjects of this study were all early-stage NSCLC patients with low malignancy, resulting in a generally ideal prognosis. However, in the comparison of SF-36 score findings, we can see that the scores of Physical Functioning, Role-Physical, and General Health were higher in the study group than in the control group, which suggests that the prognostic quality of life of our patients in the study group was better than that of the control group. This again emphasizes the excellent clinical application value of perioperative refined care.

In addition, the effect of fine surgical nursing on lung cancer was recently mentioned in a study by Dong J et al. [30]. First, it is safe to assume that the study by Dong J et al. [30] also explored the effects of fine surgical nursing on lung cancer. However, the focus of attention in the study by Dong J et al. [30] was mainly on subjective scores; for example, they looked at VAS, PSAQI, SAS/SDS, and satisfaction with care. Our study focused more on changes in objective clinical indicators, such as T-lymphocyte subsets, inflammatory factors, and prognostic survival. Meanwhile, the number of cases in our study was significantly larger. Therefore, our study can provide a more objective and comprehensive clinical reference opinion.

## Conclusion

Perioperative refined nursing for NSCLC patients undergoing thoracoscopic radical surgery can improve postoperative immunity and inhibit inflammation. It gives patients a more reliable guarantee for their postoperative rehabilitation and prognosis, which is recommended for clinical popularization and use. In the follow-up, we need to analyze more clinical objective indicators to evaluate the impact of perioperative refined care on NSCLC and increase the number of cases to improve the representativeness and comprehensiveness of the study.

## Dodatak

### Ethical approval

The study protocol was approved by the Ethics Committee of the Affiliated Hospital of Nantong University (Approval No: 2022-L144).

### Consent to publish

All authors gave final approval of the version to be published.

### Availability of data and materials

The data used to support the findings of this study are available from the corresponding author upon request.

### Funding

The authors declare that no funds, grants, or other support were received during the preparation of this manuscript.

### Acknowledgements

Not applicable.

### Conflict of interest statement

All the authors declare that they have no conflict of interest in this work.
